# The Burden of Pediatric Visual Impairment and Ocular Diagnoses in Barbados

**DOI:** 10.3390/ijerph20166554

**Published:** 2023-08-10

**Authors:** Kirsten Da Silva, Michelle Dowell, Eleonore J. Savatovsky, Dawn Grosvenor, David Callender, Michael H. Campbell, Ian Hambleton, Elizabeth A. Vanner, Alana L. Grajewski, Ta Chen Chang

**Affiliations:** 1 Bascom Palmer Eye Institute, University of Miami Miller School of Medicine, Miami, FL 33136, USA; kirstendasilva1@gmail.com (K.D.S.); esavatovsky@med.miami.edu (E.J.S.); eav45@med.miami.edu (E.A.V.); agrajewski@med.miami.edu (A.L.G.); 2 Faculty of Medical Sciences, The University of the West Indies, Cave Hill Campus, Bridgetown BB11000, Barbados; michelle.dowell@mycavehill.uwi.edu (M.D.); dawn.grosvenor@cavehill.uwi.edu (D.G.); david.callender@cavehill.uwi.edu (D.C.); michael.campbell@cavehill.uwi.edu (M.H.C.); ian.hambleton@cavehill.uwi.edu (I.H.)

**Keywords:** amblyopia, pediatrics, visual impairment, vision, blindness, refractive error, strabismus, public health, Caribbean

## Abstract

Visual impairment (VI) negatively affects a child’s quality of life. The prevalence of VI in the Caribbean is nearly three times higher than in the United States, but the causes remain uncertain. This study leverages Barbados’ unique eye care system to survey the eye diseases and VI prevalence in Barbadian children. Medical records of all patients aged <19 years who received ophthalmic care in Barbados’ two public eye care centers between January and December 2019 were reviewed, capturing the entirety of public pediatric eye care within the study period. Age at the first visit to the clinic and at the final visit in 2019, sex, best-corrected visual acuity (BCVA), past medical history, and clinical diagnoses were extracted and analyzed. VI was defined as a BCVA of 6/12 or worse in the better-seeing eye. There were 3278 patient records with a mean age at the first visit of 7.8 ± 3.9 years. There were 80 (2.4%) children with VI, 62.5% of which were attributed to amblyopia. A total of 94% of VI was preventable or treatable. The most common diagnoses were refractive error (87.5%), strabismus (27.5%), and allergic eye disease (20.0%). Amblyopia is the major cause of pediatric VI in Barbados and is largely avoidable.

## 1. Introduction

Pediatric visual impairment (VI) affects a child’s quality of life and education, as 80% of learning is typically obtained through sight [1,2]. It also shapes the transition into adulthood and negatively affects employment and social prospects [1]. Worldwide, 239 million children live with poor vision, 1.4 million are blind from diseases such as retinal and corneal disorders [2], and another two million are categorized as blind due to uncorrected refractive error [3]. Approximately 40% of childhood blindness is avoidable, meaning that the causes are preventable or that vision can be restored with treatment [3].

The causes of VI vary significantly between countries [4] and are largely determined by the country’s socioeconomic development and capacity to provide health care [3]. In high-income countries, such as the United States, the leading causes of childhood blindness are cortical visual impairment, retinopathy of prematurity (ROP), and optic nerve hypoplasia [3]. This contrasts with low-income countries, where the main causes are vitamin A deficiency and ophthalmia neonatorum [3].

The estimated prevalence of blindness in the Caribbean is nearly three times higher than in the United States [4], yet there is a paucity of data on the causes of pediatric VI within Caribbean countries. In Suriname, the leading causes of pediatric VI and blindness are ROP, retinal dystrophies, and cataract [5], and a 1988 study revealed congenital rubella syndrome as the leading cause of childhood blindness in Jamaica [6]. Through the Expanded Program on Immunization, Jamaica had eliminated rubella by 2000 [7]. With similar improvements in health care and decreasing infant mortality rates across the Caribbean [8,9], the leading causes of VI in the region have likely changed over time. Population-based data are needed to elucidate these causes. There are no data published to date on the causes of pediatric VI in Barbados.

**Figure 1:**
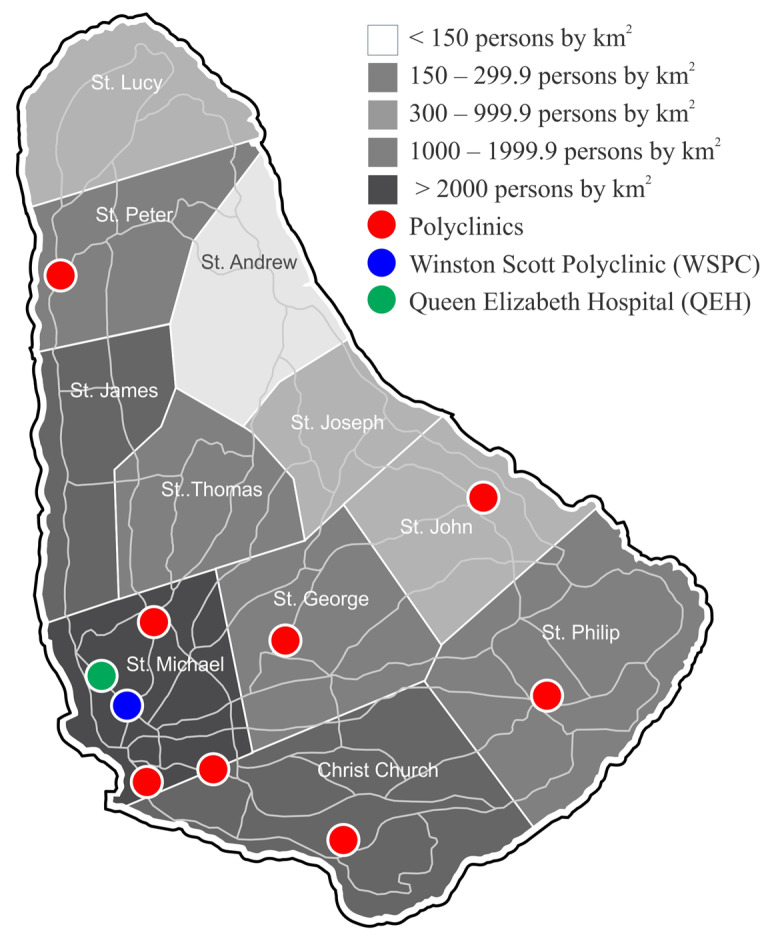
Map of Barbados showing population density by parish, location of polyclinics, Queen Elizabeth Hospital and Winston Scott Polyclinic, and road infrastructure.

Barbados is an island nation located in the eastern Caribbean with a population of more than 287,000 inhabitants on 430 square kilometers of land. Administratively, the island is divided into eleven parishes. Overall, there is a high population density with more than 668 people per square kilometer [10], and most of the population lives in the southern and western parishes. The island is classified by World Bank as a high-income country with one of the highest per capita incomes in the region [11,12]. Demographically, 92% of the population are of Afro-Caribbean descent, 3% each mixed or Caucasian, and the remainder are unspecified [11]. Approximately 21% of the population is under the age of 18 years [12]. Additionally, the island has well-developed roadway and highway infrastructure and a reliable public transportation system, making travel within the country accessible (Figure 1).

Healthcare in Barbados is free at the point of delivery in the public health sector. This is funded by the Ministry of Health and Wellness and comprises a single hospital (Queen Elizabeth Hospital [QEH], a tertiary care hospital including a Department of Ophthalmology), and nine primary care polyclinics (including the Winston Scott Polyclinic [WSPC], which has a pediatric ophthalmology service) (Figure 1).

In Barbados, the incidence and prevalence of pediatric VI is unknown. All public sector pediatric eye care is carried out at the island’s two public eye care centers at QEH and WSPC, and is attended by a single pediatric ophthalmologist, D.C., at weekly clinics at each site. The records from these two sites reflect the entirety of the Barbadian public pediatric eye care burden and outcomes. Barbadian children enter the eye care system through several avenues, including self-referral/walk-in appointments, a referral from other health care facilities, interdepartmental referrals within the QEH, or referral through the Schools Eye Service (SES). The SES is a program established by the Ministry of Health and Wellness that allows teachers and caregivers to refer students suspected of having visual problems through the school to the ophthalmology service at WSPC. Typically, younger children, children with more severe eye disease, and those requiring closer follow-up attend the eye clinic at the QEH, whereas older children with less severe eye diseases such as refractive errors and allergic eye disease are seen within the WSPC. The provision of corrective lenses is free of cost to all Barbadian citizens within the public health system. All schools on the island have access to this service. At WSPC, there is an optometrist and orthoptist available daily. Children are seen in pediatric eye clinics until they reach the age of 18 years; from the age of 19 years, they are transferred to adult eye clinics. Ongoing efforts are being made to strengthen the health information system. However, during the study period, there was no established electronic medical records system in the public sector. This study utilizes Barbados’ unique setup to perform a cross-sectional review of patient records to determine the patterns of pediatric eye diseases and VI.

## 2. Materials and Methods

The University of the West Indies Cave Hill Campus/Barbados Ministry of Health and Wellness Institutional Research Board granted ethical approval prior to the study (Reference No: 210902-B). All protocols adhered to the tenets of the Declaration of Helsinki.

Records of all patients under 19 years of age who sought ophthalmic care at the QEH and WSPC pediatric ophthalmology clinics between 1 January and 31 December 2019 (pre-COVID-19 pandemic) were manually retrieved and reviewed. Origin of referral for the first visit, age at first presentation to either clinic, age at the final visit in 2019, sex, best-corrected visual acuity (BCVA) at the final visit in 2019, past medical history, and clinical diagnoses present in 2019 were extracted from the medical records.

The clinical diagnoses were grouped by disease entity and, where appropriate, into anatomical categories: orbital and adnexal diseases, conjunctival disease, corneal disease, allergic eye disease, uveitis, cataract, refractive error, strabismus, amblyopia, ocular trauma, glaucoma-related diseases, retinal diseases, non-glaucomatous optic nerve anomalies, and miscellaneous. In cases where visual acuity was not documented at the final visit in 2019, the previous BCVA was recorded if conducted within the prior three months. If there was no documented BCVA within the prior three months, it was recorded as missing.

Patients were grouped by age into preschool (0 to <5 years), school age (5 to <12 years), and older children (12 to 18 years). Visual impairment was defined in accordance with the World Health Organization definition as a BCVA of 6/12 or worse in the better eye, and was grouped by severity into mild, moderate, severe, and blindness [13] (Table 1). Data were collected and stored using a Research Electronic Data Capture (REDCap) database at https://caribdata.org/redcap/ (accessed on 21 March 2022), which complies with Barbados Data Protection Act. Analysis was conducted by a biostatistician (E.A.V.) using SAS version 9.4 software (SAS Institute, Cary, NC, USA).

## 3. Results

Of the 3504 pediatric patients on the visit logs during 2019, 3278 (93.6%) were included for analysis. The remaining 226 were excluded because patients were 19 or older, records were not available, or patients did not attend the visit.

The mean age at first visit was 7.8 ± 3.9 years. There were 1438 males (43.9%) and 1840 females (56.1%), resulting in a male-to-female ratio of 1:1.3. School-age children (aged 5 to <12 years) comprised the largest group of patients (67.9%). There was a female predominance in all three age groups. Records reflected 4709 total visits by the 3278 patients.

A total of 589 (18%) children were seen at QEH and 2689 (82%) at WSPC, with 71 having visited both clinics during the study period. QEH patients presented at a significantly younger age than those of WSPC (3.9 ± 4.6 years vs. 8.7 ± 2.7 years, respectively; *p* < 0.0001). The main sources of initial referrals to QEH were from within the QEH (interdepartmental) and polyclinics, whereas the Schools’ Eye Service (SES) contributed most of the referrals (44.8%) to WSPC. The referral source was unknown or undocumented for 1117 children, with 1065 of these visits being made to WSPC. Table 2 shows the origin of the initial referral to the eye clinics for each child seen in 2019.

Refractive error was the most common diagnosis (45.6%) followed by allergic eye disease (34.4%), strabismus and amblyopia combined (10.1%), and adnexal diseases (2.2%). Five hundred and ninety-five (18%) children had more than one diagnosis documented in their records. Supplemental Table S1 shows the diagnoses recorded for children during 2019.

Of the 3278 children included in the dataset, 767 (23.4%) had normal ocular exam findings. The reasons for their visits included screening for refractive error, diabetic and/or sickle retinopathy, and Retinopathy of Prematurity (ROP). Visual behavior was recorded instead of visual acuity for 116 patients who were preverbal/nonverbal and/or intellectually disabled. Of these, 113 children were able to fixate and follow visual stimuli in at least one eye, two reacted to light in at least one eye, and one did not react to light in either eye. Visual acuity data was missing from the notes for an additional 126 children. Both groups of children were excluded from the visual assessment analyses.

### Visual Impairment

Visual impairment was observed in 80 (2.4%) children, with most from within the school-age group (Table 3). There were 42 (52.5%) females and 38 (47.5%) males. Of these, 62 had mild VI, 16 had moderate VI, and 2 were blind. No children had severe VI or blindness.

A total of 70 (87.5%) of the 80 children with VI had a refractive error, and 50 (62.5%) had amblyopia documented as a diagnosis in their medical records (Table 4). Of the 70 visually impaired children with refractive error, 43 (61.4%) were school age or older. Of the school-aged children, 25 (58.1%) were referred through the SES, 3 were referred from other polyclinics, and the referral source was unknown for the remaining 15. Children in the preschool age group were referred through polyclinics, private, non-pediatric ophthalmologists, or interdepartmental referrals from QEH. The referral source was unknown for three children in this age group.

## 4. Discussion

To our knowledge, this is the first study investigating the causes of pediatric VI and eye diseases in Barbados. It addresses the need for epidemiologic data on pediatric VI by revealing the common ocular diseases and causes of VI in Barbadian children as well as the need for an optimized health medical information system. This study complements the previous work undertaken by the Barbados Eye Study in the adult population [14].

Refractive error and/or refractive amblyopia were the most common diagnoses observed in visually impaired children, mirroring worldwide trends [3]. It has been established that supplying eyeglasses to visually impaired children improves their vision to within normal limits in 95% of cases [3]. Furthermore, correction of refractive error before the age of 30 months provides a higher likelihood of reaching 20/20 vision than those older than 30 months [15]. Improved academic performance may also be linked to an improvement in vision. In a randomized control study conducted in western China by Ma et al., the provision of free glasses to children was shown to improve academic performance [16]. Given that most of the visually impaired children in our study had a refractive error, early detection of high refractive error and provision of corrective lenses are public health interventions that may reduce the number of children with VI.

Allergic eye disease was another leading diagnosis seen in Barbadian children. This is unsurprising as the global prevalence of ocular allergic disease has been increasing over recent decades [17]. Although not a common cause of VI, ocular allergy can negatively impact the quality of life and school performance through absenteeism if not adequately treated [18], and it also predisposes children to keratoconus through frequent eye rubbing [19]. The pediatric ophthalmology clinic at WSPC is well equipped to manage these cases, and there is a strategic placement of a Pentacam imaging device at this clinic to allow for precise and efficient detection of keratoconus through advanced imaging techniques.

Furthermore, strabismus was another leading diagnosis and cause of VI in Barbadian children. Globally, strabismus affects between 0.14% to 5.65% of the pediatric population and remains an important cause of amblyopia and visual impairment [20]. The impact of early intervention and prompt treatment on reducing visual impairment has been well documented. Studies have shown that early detection and appropriate management with corrective lenses, occlusion therapy, or surgical intervention can improve visual outcomes and reduce long-term visual impairment associated with strabismus [21]. Similar to refractive errors, public health interventions that involve screening for the misalignment of the eyes and prompt referral for treatment can lower the prevalence of visual impairment from strabismus.

It is interesting that ROP was not a cause of visual impairment in this pediatric cohort. There were no cases of ROP detected in visually impaired children despite a reduction in Barbados’ infant mortality rate over the years [22]. This contrasts with global epidemiological data that notes ROP as the leading avoidable cause of childhood blindness in high-income countries and in middle-income countries (where neonatal care has improved but ROP management infrastructure lags behind) [3,23]. Our finding contrasts with the situation in Suriname, another Caribbean country for which data on pediatric visual impairment has been reported: ROP is one of the leading causes of VI and blindness in Surinamese children [5]. In contrast to Barbados, Suriname is a large middle-income country whose infant mortality rate stands at 16.1 per 1000 live births [24] compared to Barbados’ 9.3 per 1000 [22]. This suggests that, even with lower infant survival rates, more premature births result in ROP in Suriname. The reason for this observation is unknown, and future research should be conducted to assess the differences in factors that influence the prevalence of ROP among Caribbean countries. In our cohort, some children, especially those with normal eye examinations during the study period, were referred specifically for ROP evaluation, so the absence of a referral network is not likely to be the cause of this absence of ROP. We cannot discount the possibility that perhaps Barbadian children who developed ROP may have had dichotomous outcomes: near-normal vision from spontaneously regressed disease or complete blindness. In either case, these children may not have sought eye care and thus would not be captured in our study.

Overall, the majority of VI observed was due to avoidable causes, and most of these children had refractive error and amblyopia. Since Barbadians receive health care free at the point of delivery in the public health care system, the barriers to care likely exist elsewhere. The majority of those in our cohort with refractive error and/or refractive amblyopia first presented when they were almost eight years old (school-age), which suggests that a program that screens for high refractive error before children enter school could capture many of these children, provide the appropriate refractive correction, and decrease this VI burden.

The referral pattern on the island supports this notion. Most affected children were older and were referred through the SES to the WSPC, suggesting that earlier screening may have resulted in interventions sooner than occurred. The United Kingdom National Screening Committee recommends childhood vision screening between the ages of four and five years [25]. The United States Preventive Services Task Force also recommends that preschool vision screening occurs between three and five years of age to best detect amblyopia and its risk factors [26]. Additionally, the American Academy of Ophthalmology, the American Academy of Pediatrics, and the American Association for Pediatric Ophthalmology and Strabismus have jointly emphasized the importance of vision screening below the age of three [27]. Leon et al. found that amblyopia frequently developed in untreated patients with anisometropia below the age of three, and early treatment proved effective in reducing the development of amblyopia [28]. In addition, Longmuir et al. showed that children with hypermetropia were more likely to develop amblyopia and strabismus if left untreated until after the age of three [29].

The American Academy of Pediatrics recommends routine screening for structural abnormalities using red reflex testing in infants [30], and instrument-based screening with photoscreeners and autorefractors in children from age one until they can read an eye chart [31]. In the current Barbadian system, children aged three to five years who had both VI and refractive error would only be assessed if they had severe bilateral disease to demonstrate VI behavior, the financial means to seek vision screening with private care (for an asymptomatic child), or if they were already in the public health system for other medical reasons and had undergone vision screening for related medical reasons. Since the SES provided a large portion of the referrals in the VI group, it may also be beneficial to optimize and expand this public service to all polyclinics. In other countries, such as the United States and low- and middle-income countries, vision screening programs have been incorporated into the education sector and co-managed by the country’s Ministries of Education and Health [32]. Involving Barbados’ Ministry of Education in the development and execution of the SES may help to streamline this service and make it a more efficient route of referral. This may include formal training of teachers to detect signs of visual difficulties and to promote visual health education to students as well as provision of a school nurse to conduct visual assessments [33].

A publicly available preschool vision screening program may facilitate early detection and treatment of visual pathology and prevent amblyopia, reducing the overall prevalence of pediatric visual impairment in Barbados. Similar interventions have been successful in other countries. For example, the implementation of a preschool vision screening program using visual acuity testing in Sweden led to a reduction in the prevalence of amblyopia [34]. Several other vision screening approaches have been studied, including retinoscopy, photoscreening, stereopsis assessment, and color vision testing [35,36]. Screening at preschools, day-care centers, and community health centers can ensure easy accessibility to children and their parents, and can be carried out by trained personnel, such as nurses, orthoptists, or trained lay people [36].

Another concept that may be feasible is to attach vision screening to childhood immunization clinics. This model proved to be effective in low-resource settings, such as Nigeria, where community health workers performed visual assessments using photoscreeners and ophthalmoscopes, and incorporated visual-related screening questions into the interviews with parents at routine well-child/immunization visits [37]. In Barbados, polyclinics provide a great opportunity for children to receive vision screening, given their well-established Child Health clinic infrastructure, good distribution across the island, and easy access to Barbadians. The public health nurses working within the child health clinics may incorporate vision screening into their routine well-child assessments.

Furthermore, piggybacking off this well-established program may be cost effective and time efficient, and screening can begin in a timely manner. It would also provide multiple opportunities for screening to occur before children enter school. Alternatively, offering vision screening at schools to children entering reception/kindergarten is another option that would allow early detection of vision problems and prompt intervention. However, this model may be less convenient as it would require trained personnel to visit each school, and children would have only one opportunity for screening. Therefore, some will run the risk of not receiving screening at all.

Overall, a model that provides Barbadian children with public access to visual assessment before they enter school is essential to circumvent the existing socioeconomic barriers and ensure equitable delivery of visual health care. Additionally, it should take advantage of Barbados’ universal health care system, well-established public health facilities, and ease of travel within the country. When making decisions regarding the implementation of the screening model, careful consideration should be given to both the existing capacity of the public health workforce to accommodate the additional workload and the feasibility for the government. It should also ensure adequate follow-up and the provision of appropriate interventions and services, such as low vision services and visual rehabilitation services.

The study has several limitations. First, the data were collected solely from public eye care facilities, and data from children seen exclusively in private, non-pediatric facilities and from the School for the Blind would not have been captured, although this does not alter our evaluation of the VI and ophthalmic disease burden on the publicly funded health care system. This limitation is mitigated by the fact that there were no fellowship-trained pediatric ophthalmologists in Barbados outside of QEH and WSPC during the study period, so any child diagnosed with eye disease at a private facility would likely have been referred to QEH/WSPC and be captured in our study. Second, despite a comprehensive review of medical records, a third of the study cohort did not have a documented referral source, which precludes a more granular analysis of referral patterns. However, because most of these referrals were made to the WSPC, it could be reasonably assumed that many of these were made by the SES. Nevertheless, as the SES referral contributed to nearly half the care burden within the public eye care system, an expanded screening program such as SES targeting younger children would likely capture a large proportion of those at risk of VI. Furthermore, the absence of visual acuity data may have influenced the accurate determination of visual impairment prevalence in Barbados. Therefore, it is crucial to prioritize efforts in documenting and recording examination findings appropriately. Lastly, our study period consists of one single year, and we cannot comment on variations in disease and VI prevalence over a longer period.

## 5. Conclusions

Among Barbadian children, refractive error and/or refractive amblyopia are the major causes of VI, with refractive error and strabismus being the two most prevalent ocular diagnoses. Pediatric VI in Barbados is largely avoidable. Barbadians have the advantage of free health care and a good distribution of primary public medical facilities across the island, circumventing some of the common barriers to health care faced by many countries. This provides the island with a unique opportunity to optimize its Child Health system and establish a comprehensive Child Health program that is co-managed by the Ministries of Health and Education. This would include preschool vision screening, parent, teacher and community education on eye health, a robust health medical information system, and appropriate intervention services. This may, in turn, lower the prevalence of VI on the island.

## Figures and Tables

**Table 1:** World Health Organization categorization of Visual Impairment [13].

World Health Organization Category of Visual Impairment	Best Corrected Visual Acuity in the Better Eye
Normal Vision	Better than 6/12
Mild visual impairment	6/12–6/18
Moderate visual impairment	<6/18–6/60
Severe visual impairment	<6/60–3/60
Blindness	Worse than 3/60

**Table 2:** Origins of first referral to Barbados’ public pediatric eye clinics for children seen in 2019.

Origin of First Referral to Eye Clinic	Number of Children (n)	Percentage (%)
Schools Eye Service	1467	44.8
Interdepartmental (QEH) referral	292	8.9
Polyclinic referral	201	6.1
Private pediatrician	98	2.9
Self-referral	48	1.5
Private ophthalmologist	36	1.1
Accident and Emergency Department (QEH)	19	0.6
Unknown/undocumented	1117	34.1
Total	3278	100.0

**Table 3:** Severity of visual impairment and distribution by age group among Barbadian children.

WHO Category for Visual Impairment	Number of Children (n)	Percentage (%)
Mild	62	77.5
Moderate	16	20.0
Severe	0	0
Blindness	2	2.5
Total	80	100.0
Age Group		
Preschool (0–<5)	31	38.75
School age (5–<12)	42	52.50
Older children (12–18)	7	8.75
Total	80	100.00

**Table 4:** Diagnoses recorded for children with visual impairment. (Children may have had more than one diagnosis recorded).

Disease Entity/Diagnosis	Number of Children (n)
Refractive Error *	70
Strabismus *	22
Allergic Eye Disease *	16
Amblyopia	50
Corneal Disease *:	
● Keratoconus	2
● Dry eye disease	1
● Corneal abrasion	1
Glaucoma *:	
● Primary open angle glaucoma	3
● Juvenile open angle glaucoma	1
Cataract *:	
● Congenital cataract	2
Uveitis *:	
● Traumatic iritis	1
Retinal Diseases:	
● Retinitis pigmentosa	1
Other:	
● Nystagmus	3
● Bilateral aphakia *	1
● Ectopia lentis *	1
● Unknown	1

* Avoidable causes of visual impairment [3].

## Data Availability

The data presented in this study are available in the body of the manuscript and in the Supplementary Table S1.

## Supplementary Materials

The following supporting information can be downloaded at: https://www.mdpi.com/article/10.3390/ijerph20166554/s1, Table S1: Ocular diagnoses observed in all children seen in the public sector of Barbados during 2019.
