# Supplementary material for: The Burden of Pediatric Visual Impairment and Ocular Diagnoses in Barbados
Source: Int J Environ Res Public Health. 2023 Aug 10;20(16):6554. doi: 10.3390/ijerph20166554 (PMC10454530; doi:10.3390/ijerph20166554)
Supplement: Supplementary file 1 [file ijerph-20-06554-s001.zip › ijerph-2537491-supplementary.pdf]

**Supplementary Materials**

Table of Contents

**Table S1.** Ocular diagnoses observed in all children seen in the public sector of Barbados during 2019.

| | Total number of children in study population = 3,278 |
|---|---|
| **Ocular Diagnoses** | **Number of children (n)** |
| **Refractive error** | 1496 |
| **Allergic eye disease** | 1129 |
| **Strabismus** | 194 |
| **Amblyopia** | 136 |
| **Adnexal diseases** | |
| - Chalazion/hordeolum | 31 |
| - Ptosis | 23 |
| - Nasolacrimal duct obstruction | 2 |
| - Other | |
| o Sebaceous cyst | 6 |
| o Ectropion | 1 |
| o Lid abrasion | 1 |
| o Dermatitis | 2 |
| o Pyogenic granuloma | 1 |
| o Preseptal cellulitis | 1 |
| o Nevus | 1 |
| o Hemangioma | 1 |
| o Unknown | 1 |
| Total | 71 |
| **Glaucoma-related diseases** | |
| - Glaucoma suspect | 31 |
| - Primary Congenital Glaucoma | 7 |
| - Juvenile Open Angle Glaucoma | 2 |
| - Peters Anomaly | 1 |
| - Secondary glaucoma | |

| | |
|---|---|
| o Post- surgery | 2 |
| o Post-hyphema | 1 |
| o Angle recession | 1 |
| Total | 45 |
| **Non-glaucomatous optic nerve anomalies** | |
| - Physiologic cupping | 31 |
| - Optic nerve drusen | 2 |
| - Other | 8 |
| Total | 41 |
| **Corneal disorders** | |
| - Corneal scar | 8 |
| - Dry eye disease | 8 |
| - Keratoconus | 6 |
| - Corneal abrasion | 4 |
| - Other | 1 |
| Total | 27 |
| **Ocular trauma** | 19 |
| **Cataract** | |
| - Congenital | 9 |
| - Steroid-induced | 3 |
| - Traumatic | 1 |
| - Other | 6 |
| Total | 19 |
| **Retinal diseases** | |
| - Retinopathy of prematurity | 2 |
| - Retinitis pigmentosa | 2 |
| - Other | |
| o Chorioretinal scar | 2 |
| o Chorioretinal coloboma | 1 |
| o Best disease | 1 |
| o Stargardt disease | 1 |
| Total | 9 |
| **Uveitis** | |
| - Post-trauma | 5 |
| **Non- allergic conjunctival disease** | |

| | |
|---|---|
| - Conjunctival abrasion | 4 |
| - Subconjunctival hemorrhage | 2 |
| - Conjunctival nevus | 1 |
| - Chemical conjunctivitis | 1 |
| - Redundant conjunctiva | 1 |
| - Pterygium | 1 |
| Total | 10 |
| **Orbital disease** | |
| - Orbital cellulitis | 2 |
| **Non-organic visual symptoms** | 39 |
| **Other** | |
| - Congenital idiopathic iris atrophy | 1 |
| - Iris nevus | 4 |
| - Iris coloboma | 1 |
| - Iris and retina coloboma | 1 |
| - Nystagmus | 1 |
| - Color vision deficiency | 1 |
| - Ocular migraine | 4 |
| - Bell's palsy | 2 |
| - Physiologic diplopia | 1 |
| Total | 16 |
| **Unknown** | 5 |
